# The Versatile Role of GDF5 in Chondrogenic Progenitor Cell-mediated Cartilage Regeneration via a Hyaluronic Acid-fibrin IPN Hydrogel Platform

**DOI:** 10.1007/s11095-026-04079-3

**Published:** 2026-04-14

**Authors:** Rui He, Venkateswaran Ganesh, Pornpoj Phruttiwanichakun, James A. Martin, Dongrim Seol, Aliasger K. Salem

**Affiliations:** 1 Pharmaceutical Sciences and Experimental Therapeutics, College of Pharmacy, University of Iowa, 180 S Grand Ave, Iowa City, IA 52242 USA; 2 Department of Orthopedics and Rehabilitation, Carver College of Medicine, University of Iowa, 200 Hawkins Dr, Iowa City, IA 52242 USA; 3 Roy J. Carver Department of Biomedical Engineering, College of Engineering, University of Iowa, Iowa City, IA USA; 4 Department of Orthodontics, College of Dentistry and Dental Clinics, University of Iowa, Iowa City, IA USA

**Keywords:** cell homing, chondrogenic differentiation, chondrogenic progenitor cell, GDF5, IPN hydrogel

## Abstract

**Objective:**

Focal damage to articular cartilage incurred during joint injuries frequently progresses to post-traumatic osteoarthritis (PTOA) due to the limited intrinsic repair capacity of cartilage. Chondrogenic progenitor cells (CPCs) residing within the cartilage can contribute to repair if effectively recruited and activated. Early interventions that enhance CPC homing and their subsequent chondrogenesis offer a regenerative strategy to prevent PTOA progression, addressing the current lack of effective early clinical therapies. GDF5 stands out as a key protein involved in cartilage development, yet its potential to mobilize CPC-mediated regeneration remains underexplored.

**Methods:**

We evaluated the effects of GDF5 on CPC migration, proliferation, chondrogenic differentiation, and anti-catabolic activity using *in vitro* CPC models. To assess CPC chemotaxis in a clinically relevant biomaterial context, GDF5 was incorporated into a hyaluronic acid/fibrin interpenetrating network (IPN) hydrogel and tested in an ex vivo cartilage defect model.

**Results:**

GDF5 acted as a potent chemoattractant for CPCs, promoting their recruitment toward cartilage defects when delivered via a hyaluronic acid/fibrin IPN hydrogel in an ex vivo model. GDF5 also enhanced CPC proliferation, consistent with activation of a glycolysis-associated transcriptional program. In addition, GDF5 significantly upregulated chondrogenic markers, including SOX9, COL2a1, and ACAN, and elevated extracellular matrix components in CPCs, potentially through activation of the PI3K/AKT signaling pathway. Furthermore, GDF5 reduced expression of a key catabolic enzyme ADAMTS5, possibly through the WWP2/miR-140 axis.

**Conclusion:**

These findings highlight the versatile role of GDF5 on endogenous CPCs. When combined with a hydrogel platform, GDF5 may serve as an early therapeutic strategy to convert injured cartilage from a passive site of degeneration into one of active regeneration.

**Graphical Abstract:**

**Figure:**
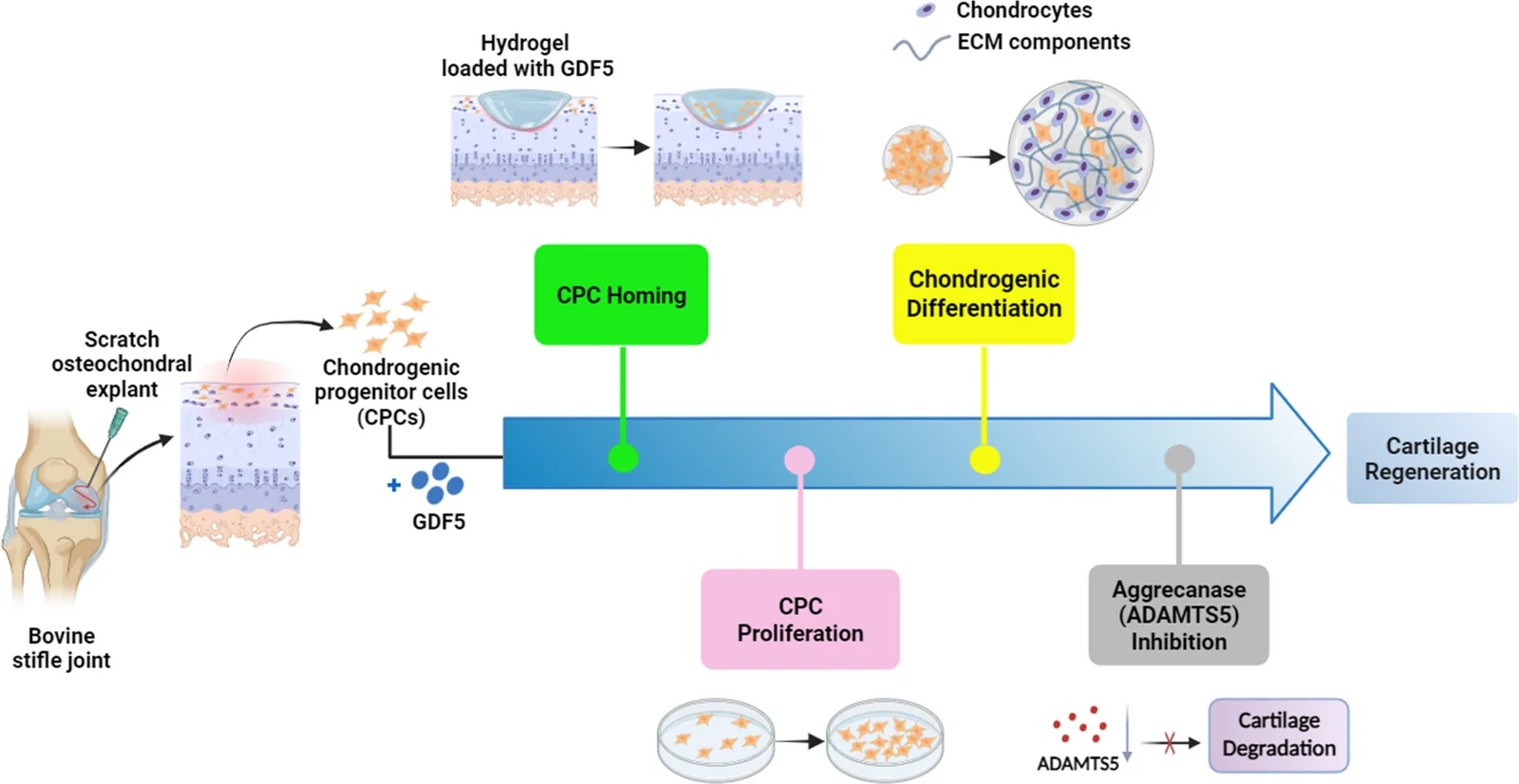

**Supplementary Information:**

The online version contains supplementary material available at https://doi.org/10.1007/s11095-026-04079-3.

## Introduction

Post-traumatic osteoarthritis (PTOA) is a distinct and increasingly prevalent form of osteoarthritis that develops following joint injury, such as anterior cruciate ligament (ACL) rupture, meniscal damage, or intra-articular fracture [1]. After joint injury, a cascade of inflammatory and mechanical changes occurs, leading to cell death, loss of extracellular matrix (ECM), and gradual breakdown of cartilage tissue. PTOA often affects younger individuals and can lead to long-term joint dysfunction [2, 3]. PTOA accounts for approximately 12% of osteoarthritis cases, affecting over 5 million people in the U.S., and up to 50% of ACL injury patients develop PTOA even after reconstruction [3, 4]. Current clinical treatments primarily manage symptoms rather than addressing the underlying pathology. Non-surgical interventions, including nonsteroidal anti-inflammatory drugs and physical therapy, provide temporary relief and improve joint function but do not prevent cartilage degeneration [5, 6]. Surgical options such as microfracture, autologous chondrocyte implantation (ACI), and osteochondral allograft transplantation (OCA) are commonly used to repair cartilage defects. However, microfracture typically produces fibrocartilage that initially fills defects but lacks the durability and mechanical properties of native hyaline cartilage [5], while ACI and OCA are more invasive, carry notable reoperation rates, and yield variable long-term outcomes [7, 8]. The well-defined onset of PTOA presents a critical opportunity for early intervention, yet such timely and potentially disease-modifying strategies remain largely underutilized in clinical practice.

Chondrogenesis has emerged as a promising approach for early-stage treatment for PTOA, offering a regenerative strategy that targets the underlying pathology of the disease. It involves the differentiation of chondrogenic progenitor cells (CPCs) into chondrocytes, the specialized cells responsible for synthesizing ECM components [9]. CPCs are a small but active population residing in the superficial zone of articular cartilage [9, 10]. In contrast to mature chondrocytes, which are metabolically limited and poorly responsive to injury, CPCs exhibit a more adaptable phenotype and retain regenerative potential [5, 9]. Following cartilage injury, CPCs become activated, proliferate, and produce essential cartilage matrix components, suggesting that therapeutic strategies could be designed to stimulate these cells *in situ* [11, 12]. Increasing interest has therefore turned toward harnessing these endogenous progenitor cells as an alternative to more invasive exogenous cell-based therapies, such as autologous chondrocyte or mesenchymal stem cell (MSC) transplantation, which face challenges such as contamination during cell expansion, poor cell survival after implantation, or immune rejection [13, 14]. By contrast, CPC-based therapies could capitalize on the cells already present in the joint, avoiding many of these barriers [9]. Moreover, advances in biomaterials, chemotactic signaling, and minimally invasive delivery platforms now make it feasible to enhance the CPC response during the early post-injury period, thus transforming damaged cartilage from a passive site of degeneration into an active regenerative interface [9, 15–17]. Among these strategies, interpenetrating network (IPN) hydrogels composed of hyaluronic acid (HA) and fibrin represent a promising platform for cartilage regeneration. HA provides bioactivity and mimics native cartilage matrix, fibrin supplies biocompatible, cell-adhesive sites, and together the IPN structure offers enhanced mechanical stability and robust support for regeneration [15, 18].

Other than chondrogenic approaches that focus on enhancing ECM synthesis, strategies targeting the inhibition of elevated catabolic enzymes and restoring homeostatic balance offer another promising therapeutic platform. In a healthy joint, cartilage homeostasis is maintained through a dynamic equilibrium between anabolic and catabolic processes. However, this homeostatic balance is disrupted in the context of PTOA. Increased mechanical stress, inflammation, and aging lead to elevated catabolism and diminished anabolism [19, 20]. This imbalance, characterized by a pro-catabolic response, leads to damage and the eventual loss of hyaline cartilage. The degradation of type II collagen and proteoglycan in the ECM is regulated by a suite of catabolic enzymes, such as matrix metalloproteinases (MMPs) and a disintegrin and metalloproteinase with thrombospondin motifs (ADAMTS) [6, 20]. Unfortunately, the inherent capacity of cartilage to counteract enzymatic damage and engage in self-healing processes remains severely constrained [21].

Growth and Differentiation Factor 5 (GDF5) belongs to the transforming growth factor-beta (TGFβ) superfamily, playing a pivotal role in various cellular processes, particularly in joint formation in the embryo [22]. The molecular pathway involves GDF5 binding to its receptor, typically bone morphogenetic protein (BMP) receptors, initiating downstream signaling cascades that activate the suppressor of mothers against decapentaplegic proteins and other signaling molecules, ultimately regulating gene expressions related to chondrogenic differentiation [22, 23]. Studies have shown that GDF5 could enhance chemotaxis and chondrogenic differentiation of bone marrow mesenchymal stem cells (BMSCs) *in vitro* [24–26], and suppress the expression of enzymes responsible for degrading the ECM in human chondrocytes, including ADAMTS4 and MMP13 [27]. Therefore, we hypothesize that GDF5 could serve as a therapeutic reagent for CPC-based cartilage regeneration by promoting chondrogenic differentiation and simultaneously exerting anti-catabolic function. However, the direct effects of GDF5 on CPCs have not been defined to our knowledge. To address this gap, we evaluated the impact of GDF5 on CPC migration, proliferation, chondrogenic differentiation, and catabolic suppression. Establishing these fundamental cell-level responses is essential for guiding the design of HA/fibrin IPN hydrogels and other biomaterial platforms that aim to use GDF5 to enhance CPC-mediated cartilage regeneration.

## Materials and Methods

### Joint Cell Isolation

Bovine CPCs from explant surfaces of stifle joint cartilage were isolated to evaluate the functional roles of GDF5 as described previously with some modifications [11]. Briefly, stifle joints were collected from young adult cattle, aged 15–24 months, at a local abattoir (Bud's Custom Meats, Riverside, IA, USA). Osteochondral explants were generated by manually sawing an approximately 35 × 35 mm portion of the bovine femoral condyle, including the central load-bearing region of the articular surface. The surfaces of osteochondral explants were scratched using a sterile 26-gauge needle. The explants were incubated in medium containing Dulbecco’s Modified Eagle Medium (DMEM; Gibco, Waltham, MA, USA)/F12 Medium (Gibco) (1:1) supplemented with 10% (v/v) fetal bovine serum (FBS; Gibco) and 1% (v/v) penicillin–streptomycin (PS; Gibco) at 37 °C, 5% CO_2_/O_2_ for 10–14 days. Bovine CPCs were collected by trypsinizing the explant surface with 0.25% (v/v) trypsin–EDTA (Gibco) for 10 min and cultured in the same medium. Passages 2 through 4 of CPCs were used in all experiments.

### Cell Migration

**Fig. 1:**
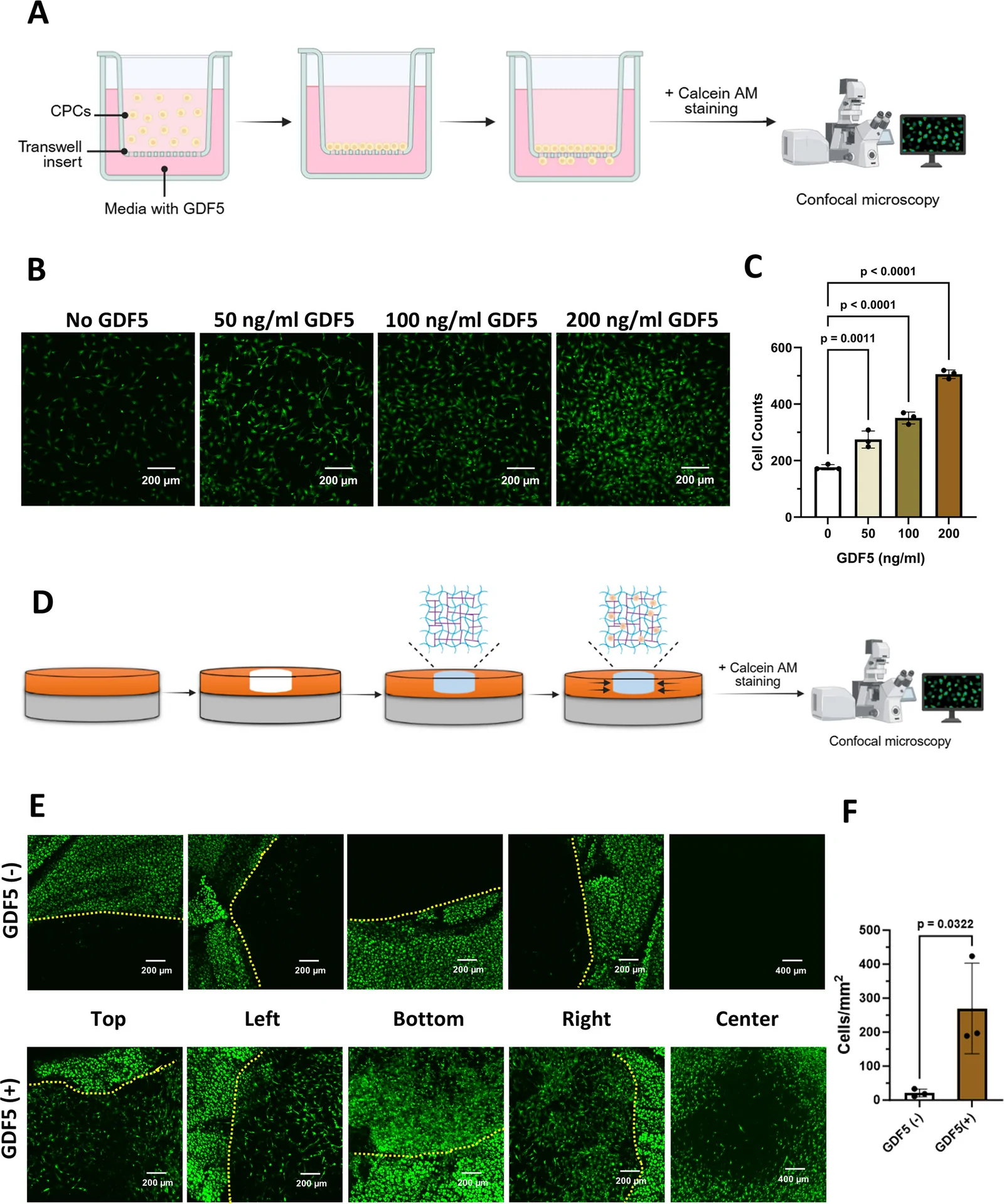
GDF5 enhances the migration of CPCs. (**A-C**) *In vitro* Transwell assay (*n* = 3): (**A**) Schematic representation of Transwell assay. (**B**) Confocal images of migrated CPCs stained with Calcein AM (green) and (**C**) quantitative analysis of migrated CPCs. (**D-F**) Ex vivo migration assay (*n* = 3): (**D**) Schematic representation of ex vivo migration assay. (**E**) Ex vivo explants with a chondral defect (dotted lines). Stacked confocal images of explants stained with Calcein AM (~ 1 mm depth and 20 µm intervals). Images were captured at 2 × magnification from the center of the defect, and at 4 × magnification showing the defect site from the top, left, bottom, and right perspectives. **(F)** Quantification of migrated cells within the defect region, expressed as cell density (cells/mm^2^). Data are represented as means ± standard deviation. For comparisons between two groups, statistical significance was assessed using an unpaired *t*-test. For experiments involving multiple groups, one-way ANOVA followed by Sidak’s post-hoc test was performed. A *p*-value less than or equal to 0.05 was considered significant.

Cell migration induced by GDF5 was assessed using a Transwell® migration assay (Fig. 1A) and an ex vivo explant model (Fig. 1D).

In the Transwell study, bovine CPCs were seeded into inserts of Transwell 24-well plates with 3.0 µm pores (Corning, Glendale, AZ, USA). Serum-free culture medium (DMEM/F12 supplemented with 1% (v/v) insulin-transferrin-selenium (ITS; Gibco), 50 µg/ml ascorbic acid (Sigma-Aldrich, Burlington, MA, USA), and 1% (v/v) PS) containing varying concentrations (0, 50, 100, and 200 ng/ml) of recombinant human GDF5 (rhGDF5; R&D Systems, Minneapolis, MN, USA) was added to evaluate its effect on cell migration. After the overnight incubation, inserts were washed twice with Hank’s Balanced Salt Solution (HBSS; Gibco) and stained with Calcein AM (Thermo Fisher Scientific, Waltham, MA, USA) in HBSS (1:1000 dilution). Unbound stains were removed with two additional washes, and the inserts were imaged using an Olympus FV1000 confocal microscope (Olympus, Center Valley, PA, USA). Fluorescent intensity was quantified using ImageJ (National Institutes of Health, Bethesda, MA, USA).

The ex vivo bovine explant model was established as previously described [15, 28]. Full-thickness chondral defects (4.5 mm in diameter and ~ 2 mm thick) were made in an osteochondral explant (~ 10 mm in diameter). Hydrogels were prepared by 1:1 ratio mixing of 0.5% (v/v) hyaluronic acid (HA; GelOne®, Zimmer, Warsaw, IN, USA)−20 U/ml thrombin (TISSEEL™, Baxter Healthcare, Westlake Village, CA, USA) (solution A) and 25 mg/ml fibrinogen (TISSEEL™) (solution B) at 4 °C, with or without 1 μg/ml of rhGDF5. Then the hydrogel was implanted into each defect, and explants were cultured in serum-free medium. On day 14, the explants were stained with Calcein AM as mentioned above, and cell migration was monitored using confocal microscopy. Migrated cells were quantified using ImageJ by averaging automated cell counts from five images acquired from different fields of view per sample.

### Cell Proliferation

To evaluate short-term CPC proliferation in response to GDF5, CPCs (8.5 × 10^3^ cells/well) were seeded in 96-well plates in 0.1 ml of serum-free medium with or without rhGDF5 (200 ng/ml). After ~ 20 h, total DNA content was quantified using the QUANT-iT PicoGreen dsDNA assay (Invitrogen, Waltham, MA, USA). Fluorescence was measured using a microplate reader (SpectraMax M5, Molecular Devices, San Jose, CA) at 480 nm excitation and 520 nm emission. In parallel, cell metabolic activity/viability was assessed using the MTS assay (Promega, Madison, WI, USA). For longer-term proliferation, CPCs (5 × 10^4^ cells/well) were seeded into 24-well plates in complete growth medium as described in Sect. "Joint Cell Isolation". After a 24-h attachment period, the medium was replaced with 0.5 ml of serum-free medium containing rhGDF5 at 0, 50, 100, or 200 ng/ml. Medium (with or without rhGDF5) was refreshed on day 4. After 7 days of treatment, DNA content was quantified using the QUANT-iT PicoGreen assay according to the manufacturer’s instructions.

### Cell Culture for Chondrogenic Differentiation

Two cell culture models were used to evaluate chondrogenic differentiation: 2-dimensional (2D) monolayer culture and 3D pellet culture. For 2D monolayer culture, 5 × 10^4^ CPCs were added to each well of 24-well plates. After a 24-h attachment period, the medium was then replaced with 0.5 ml of serum-free culture medium. Three concentrations of rhGDF5 (50, 100, and 200 ng/ml) were used for the evaluation. For 3D pellet culture, 5 × 10^5^ CPCs were added to a 15-ml tube before centrifugation at 500 g for 5 min to form a cell pellet. After 48-h incubation, the medium was replaced with 3 ml of serum-free medium further supplemented with or without GDF5. The culture medium was replaced every 3–4 days.

### Gene Expression by Quantitative Real-time Polymerase Chain Reaction (qRT-PCR)

In the 2D monolayer culture for the chondrogenic differentiation study, the total RNA was collected 2 and 7 days post the GDF5 treatment. TRIzol reagent (Invitrogen) and Direct-zol RNA kit (Zymo Research, Irvine, CA, USA) were utilized for RNA extraction according to the manufacturer’s instructions. The qRT-PCR was performed with PrimeTime One-Step RT-qPCR Master Mix (Integrated DNA Technologies, Iowa City, IA, USA) and Taqman® primer/probe sets (Applied Biosystems, Waltham, MA, USA) in a QuantStudio™ 3 System (Applied Biosystems). Actin beta (*ACTB*), glyceraldehyde-3-phosphate dehydrogenase (*GAPDH*), SRY-box transcription factor 9 (*SOX9*), collagen type II alpha 1 chain (*COL2a1*), and aggrecan (*ACAN*) primer/probe sets were used to assay gene expression. *ACTB* was used as a housekeeping gene for normalization. Twenty ng of RNA per sample was tested in duplicates. The delta-delta-Ct method was employed to calculate the relative changes in gene expression levels [29]. The details on the assayed genes are included in Table I.

**Table I:** Description of the Assayed Genes and Taqman® Primer/Probe Sets Used in QRT-PCR

Gene Symbol	Gene Name	NCBI Accession No	Taqman® Assay ID
ACTB	actin beta	NM_173979.3	Bt03279174_g1
GAPDH	glyceraldehyde-3-phosphate dehydrogenase	NM_001034034.2	Bt03210913_g1
SOX9	SRY-box transcription factor 9	XM_024981096.2	Bt07108872_m1
COL2a1	collagen type II alpha 1 chain	NM_001001135.3	Bt03251861_m1
ACAN	aggrecan	NM_173981.2	Bt03212186_m1
ADAMTS5	a disintegrin and metalloproteinase with thrombospondin-5 motif	NM_001166515.1	Bt04230785_m1

### Wet Weight of Pellets

In the 3D pellet culture, following 7 and 14 days of pro-chondrogenesis treatment, the pellets were collected and washed with phosphate-buffered saline (PBS). The excessive moisture on the pellet surface was removed, and the pellets were weighed on a balance (Mettler Toledo, Columbus, OH, USA).

### Glycosaminoglycan (GAG) Assay with Dimethyl-methylene Blue (DMMB) Stain

GAGs were assayed using a DMMB staining method as previously described [30]. For 2D monolayer culture, 7 days after the culture period, wells were washed with PBS. Samples were freeze-thawed three times after adding 100 μl of sterile water. A 200 μl solution of papain buffer (150 μg/ml papain) was added to each well and incubated at 60°C. In the 3D pellet culture, 7 and 14 days after the culture period, the pellets were collected, washed with PBS, and digested with 100 μl of papain buffer at 60°C. GAGs in the digest were measured with DMMB (Sigma-Aldrich) at an absorption of 540 nm on a plate reader (SpectraMax M5, Molecular Devices, San Jose, CA, USA). The standard curve was established using chondroitin-sulfate (Thermo Fisher Scientific). Results were normalized to the DNA content determined using the QUANT-IT PicoGreen kit.

### Three-dimensional (3D) Pellet Safranin-O (Saf-O)/Fast Green Histology

Pellets cultured for 14 days were washed with PBS and used for histology studies. Ten μm-thick sections of each sample were made and stained. Proteoglycan content was determined using cationic stain safranin-O (Saf-O; Thermo Fisher Scientific) and counterstained with fast green (Thermo Fisher Scientific). Cryosections were warmed to room temperature and placed in deionized water. The samples were then placed in 0.03% (w/v) Saf-O for 15 min. The slides were then rinsed in deionized water, followed by 3 min in 0.001% (w/v) fast green. The samples were dehydrated in increasing concentrations of ethanol and cleared in xylene before the cover-slipping. The sections were imaged in a transmitted light mode on an Olympus BX-61 microscope (Olympus, Center Valley, PA, USA).

### RNA Sequencing

The total RNA collected 7 days post the GDF5 treatment (200 ng/ml) from the 2D monolayer culture for chondrogenic differentiation was used for RNA sequencing. The determination of RNA quality and quantity, library preparation, and RNA sequencing were performed by Novogene Co., LTD (Sacramento, CA) as a service. Genes with adjusted *p*-value < 0.05 and |Log2(FoldChange)|> 1 were considered as differentially expressed. Then we performed Gene Ontology (GO) and Kyoto Encyclopedia of Genes and Genomes (KEGG) function enrichment analysis using NovoMagic platform served by Novogene.

### Inhibition of ADAMTS5

To investigate the inhibition of ADAMTS5, a CPC monolayer culture, as described in Sect. "Cell Culture for Chondrogenic Differentiation", was used, followed by RNA extraction and qRT-PCR as outlined in Sect. "Gene expression by Quantitative Real-time Polymerase Chain Reaction (qRT-PCR)" with *ADAMTS5* Taqman® primer/probe sets (Applied Biosystems). To evaluate the anti-*ADAMTS5* effect in CPCs under inflammatory conditions, CPCs were treated with rhGDF5 for 2 days, followed by the addition of 1 or 5 ng/ml of recombinant bovine interleukin-1 beta (rbIL-1β; ThermoFisher) and incubation for 24 h. Total RNA was then extracted from each group, and qRT-PCR was performed. The details on the assayed genes are included in Table 1.

### Statistics

The scatter plots expressed as the mean values with the standard deviation were prepared using GraphPad Prism (Version 10.1.2; San Diego, CA, USA). For comparisons between two groups, statistical significance was assessed using an unpaired *t*-test. For experiments involving multiple groups, one-way analysis of variance (ANOVA) followed by Sidak’s post-hoc test was performed. A *p*-value less than or equal to 0.05 was considered statistically significant. Differential expression analysis for RNA sequencing was performed using DESeq2, which models count data with a negative binomial distribution, and *p*-values were adjusted for multiple testing using the Benjamini–Hochberg method to control the false discovery rate (FDR).

## Results and Discussion

### CPC Migration

CPC chemotaxis is an essential prerequisite for repair in response to joint injury. In damaged cartilage, alarmins released from injured tissue act as endogenous signals that naturally attract CPCs to the defect site [11, 15]. GDF5 has been reported to regulate migratory behavior in several musculoskeletal cell populations, highlighting a broader role in coordinating cell recruitment during tissue repair. Previous studies have demonstrated that GDF5 can induce chemotactic responses in BMSCs and tendon- or ligament-derived cells [24, 26, 31, 32]. These findings suggest that GDF5 could function as a generalized chemoattractant within joint tissues, helping to coordinate reparative cell recruitment following injury. To investigate whether GDF5 could further enhance the chemotactic response of CPCs *in vitro*, a migration assay was conducted using a Transwell chamber. Images and statistical analyses of Calcein AM staining (Fig. 1B and C) revealed that the number of migrated CPCs was significantly higher in the GDF5-treated groups in a dose-dependent manner compared to the untreated group. Notably, cell condensations were observed in the high GDF5 concentration group, which has been shown as a prerequisite for chondrogenesis [33].

To further assess CPC migration in a more physiologically relevant context, we employed an ex vivo explant model using an IPN hydrogel scaffold. The IPN hydrogel composed of a HA network and fibrin fibers has been well established and characterized as a biocompatible scaffold for cartilage repair [15, 34, 35]. HA provides biochemical cues resembling native cartilage and maintains a hydrated, viscoelastic environment, while fibrin forms a degradable, cell-adhesive network that supports cell entry and spreading. The two networks interlock into a sturdier matrix that slows degradation, withstands mechanical loading, and retains proteins for sustained local delivery [15, 36]. When loaded with GDF5, the IPN hydrogel may amplify the natural alarmin-driven homing of CPCs, thereby enhancing recruitment into cartilage defects. In our previous work, the IPN hydrogel loaded with recombinant human stromal cell-derived factor 1 alpha (rhSDF-1α) was implanted into cartilage defects in explants and effectively recruited CPCs to the hydrogel [15]. Due to the continuous release of protein from the hydrogel [15], 1 μg/ml of rhGDF5 was incorporated into the IPN hydrogel in the present ex vivo migration study. As shown in Fig. 1E, minimal cell migration was observed at the defect edge in the control group, with most of the defect area remaining devoid of cells. In contrast, the rhGDF5-loaded hydrogel group exhibited pronounced cell migration from the surrounding cartilage toward the central region of the defect. Quantitative analysis confirmed a significantly higher (> 10-fold) density of migrated cells in the GDF5-loaded hydrogel group compared with controls (Fig. 1F). The defect boundaries were clearly delineated, owing to the distinct morphological differences between CPCs and native chondrocytes. These results demonstrate that the GDF5-loaded IPN hydrogels promote CPC homing into cartilage defects in an ex vivo setting.

### CPC Proliferation

**Fig. 2:**
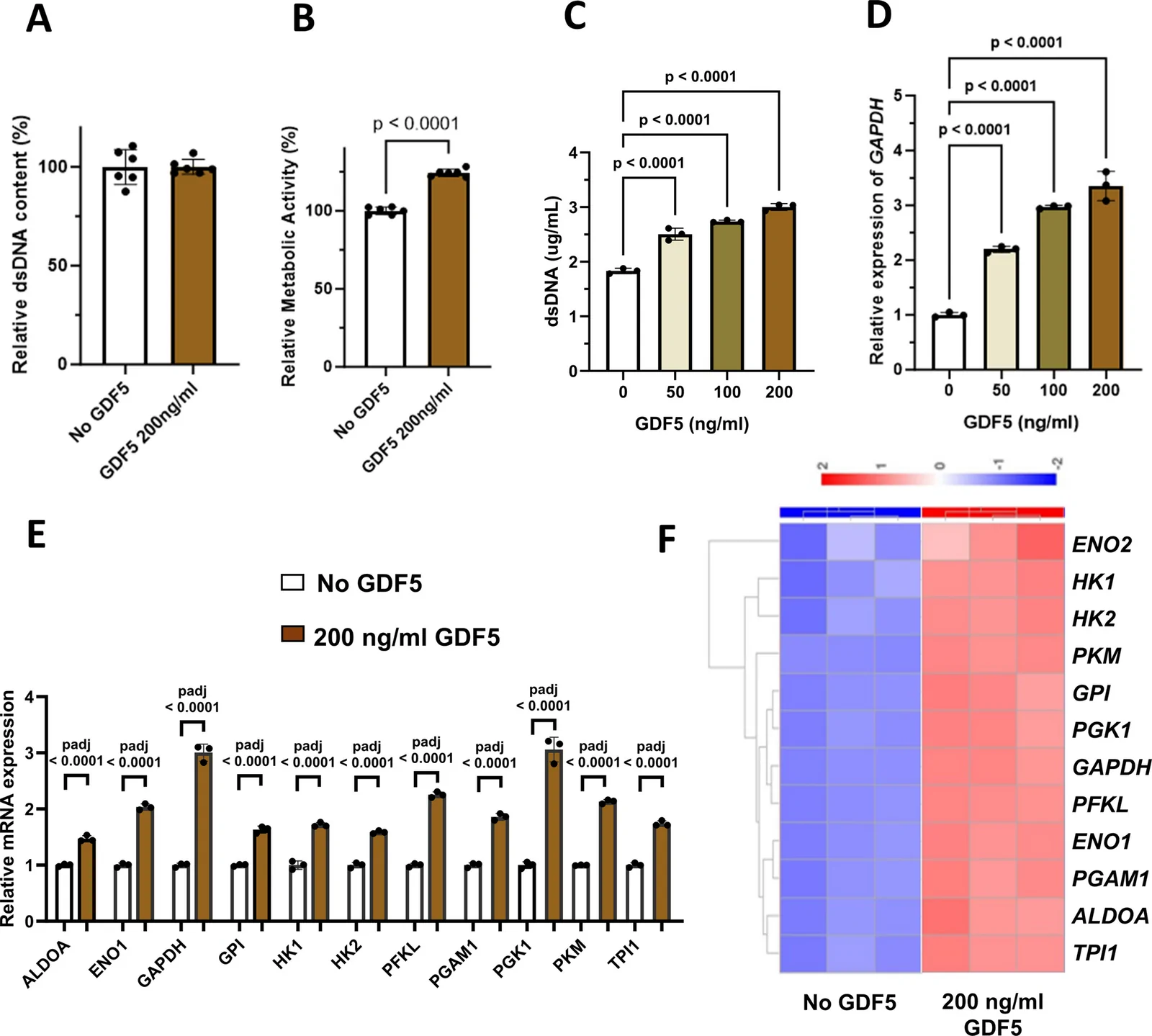
Short-term GDF5 exposure elevates CPC metabolic activity, and prolonged treatment leads to increased CPC proliferation. (**A–B**) Short-term response (~ 20 h; *n* = 6): total dsDNA content **(A)** and metabolic activity measured by MTS assay (**B**) following treatment with 200 ng/ml GDF5. (**C-F**) Long-term response (7 days; *n* = 3): (**C**) CPC proliferation via total dsDNA content following GDF5 treatment. (**D**) qRT-PCR analysis of *GAPDH*. (**E**) clustering heatmap and (**F**) quantitative comparison for key glycolysis-related genes from RNA sequencing. Data are represented as means ± standard deviation. For comparisons between two groups, statistical significance was assessed using an unpaired *t*-test. For experiments involving multiple groups, one-way analysis of variance (ANOVA) followed by Sidak’s post-hoc test was performed. A *p*-value less than or equal to 0.05 was considered significant. Differential expression analysis for RNA sequencing was performed using DESeq2, and *p*-values were adjusted (padj) for multiple testing using the Benjamini–Hochberg method to control the FDR. Padj of less than or equal to 0.05 were considered significant. *ENO2*: Enolase 2, *HK1*: Hexokinase 1, *HK2*: Hexokinase 2, *PKM*: Pyruvate kinase M1/2, *GPI*: Glucose-6-phosphate isomerase, *PFKL*: Phosphofructokinase liver type, *PGAM1*: Phosphoglycerate mutase 1, *ALDOA*: Aldolase, Fructose-Bisphosphate A, *TPI1*: Triosephosphate isomerase 1.

To assess the short-term response of CPCs to GDF5, cells were incubated with rhGDF5 for ~ 20 h, followed by a PicoGreen quantification of total genomic DNA and an MTS assay to evaluate metabolic activity. DNA content did not differ between the untreated and 200 ng/ml of GDF5-treated groups, whereas MTS values were significantly higher in the GDF5 group (Fig. 2A and 2B). This discrepancy indicates that GDF5 acutely enhances cellular metabolic activity rather than increasing cell number. When considered alongside the Transwell migration data, these findings suggest that the increase in migrated CPCs was primarily attributable to the chemoattractant effect of GDF5, while the elevated metabolic activity may reflect an early activation state that supports motility during the migration process [37].

Although short-term exposure to GDF5 did not alter cell numbers, we next examined CPC proliferation over a longer period. After 7 days of treatment, GDF5 significantly increased cell proliferation in monolayer culture with a dose-dependent pattern (Fig. 2C). Consistent with the observed trend of cell proliferation, the expression of *GAPDH*, a glycolytic marker associated with proliferative activity, was significantly upregulated upon GDF5 treatment based on the qRT-PCR analysis (Fig. 2D). Further RNA sequencing analysis revealed a global upregulation of glycolysis-related genes such as *GAPDH* and phosphoglycerate kinase 1 (*PGK1*) in CPCs treated with 200 ng/ml of GDF5 (Fig. 2E and 2 F). Gene Set Enrichment Analysis (GSEA) for KEGG further confirmed that the glycolysis pathway was significantly activated in response to GDF5 treatment (Figure S1A). These findings suggest an activation of a glycolysis-associated transcriptional program in CPCs following extended exposure to GDF5. While functional metabolic assays were not performed, the observed transcriptional changes may reflect features of metabolic adaptation to hypoxia-like conditions, similar to mature chondrocytes, which rely predominantly on glycolysis to meet their energy demands [38–40].

### Chondrogenic Differentiation

Chondrogenic differentiation is traditionally studied using 3D pellet cultures, which provide a permissive environment for cell–cell interactions, ECM synthesis, and matrix retention. More recently, 2D monolayer systems have emerged as complementary models that offer practical advantages, including ease of culture, higher throughput, and simplified RNA extraction [30]. Given these differences, we investigated the role of GDF5 in CPC differentiation using both approaches. We focus qRT-PCR analysis on the chondrogenic biomarkers, including COL2a1, ACAN, and SOX9 on the 2D monolayer culture. In parallel, we assessed the impact of GDF5 on ECM accumulation using the 3D pellet model. This was assessed by determining the wet weight of pellets, GAGs production using DMMB staining, and the abundance of proteoglycan using histological stains.

**Fig. 3:**
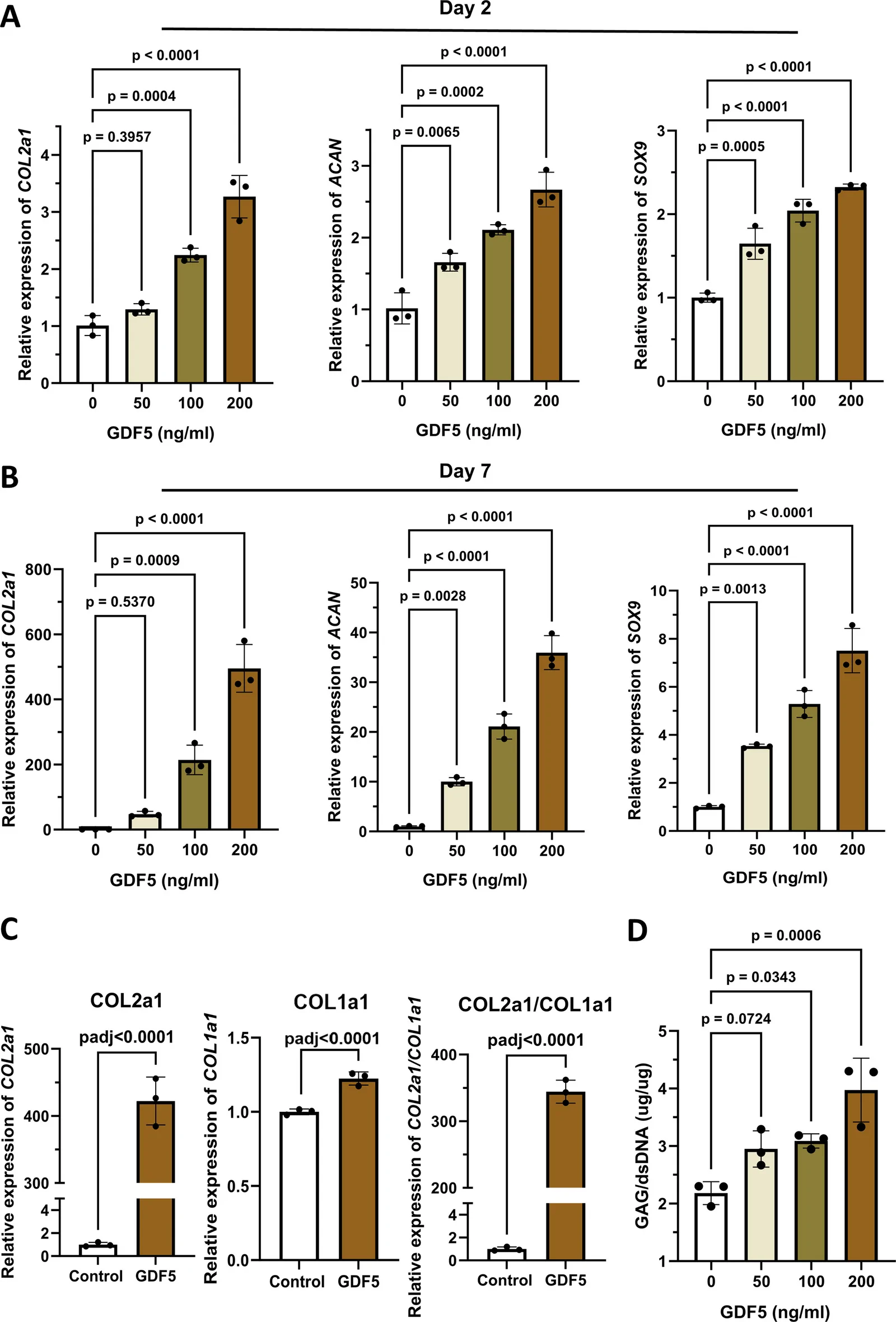
GDF5 promotes chondrogenic differentiation of CPCs in a monolayer culture model. (A and B) qRT-PCR analysis of chondrogenic markers *COL2a1*, *ACAN*, and *SOX9* treated with GDF5 at days 2 (**A**) and 7 (**B**) (*n* = 3). (**C**) RNA sequencing analysis of *COL2a1, COL1a1*, and the *COL2a1/COL1a1* ratio after 200 ng/ml GDF5 treatment at 7 days (*n* = 3). (**D**) Glycosaminoglycan (GAG) contents normalized to dsDNA following GDF5 treatment at 7 days (*n* = 3). Data are represented as means ± standard deviation. Statistical analysis was performed using one-way ANOVA, followed by Sidak’s post-hoc test. A *p*-value less than or equal to 0.05 was considered significant. Differential expression analysis for RNA sequencing was performed using DESeq2, and padj for multiple testing using the Benjamini–Hochberg method to control the FDR. Padj of less than or equal to 0.05 were considered significant.

The expression of anabolic genes, including COL2a1, ACAN, and SOX9, following GDF5 treatment in the 2D monolayer culture was depicted in Fig. 3, with Fig. 3A representing 2 days and Fig. 3B representing 7 days of treatment. A dose-dependent and rapid upregulation of these marker genes was observed by day 2 post-treatment. This effect became more pronounced over time, with *COL2a1*, *ACAN*, and *SOX9* expression levels increasing by over 400-fold, 30-fold, and 7-fold, respectively by day 7 post-treatment using 200 ng/ml of GDF5.

RNA sequencing analysis confirmed the qRT-PCR findings, revealing an over 400-fold increase in *COL2a1* expression following treatment with 200 ng/ml of GDF5 (Fig. 3C). In contrast, *COL1a1* expression increased only by 20% (Fig. 3C). The increased ratio of collagen type II to collagen type I is a well-established marker of chondrogenic differentiation and healthy chondrocytes [41]. GDF5 treatment elevated the *COL2a1/COL1a1* ratio by approximately 350-fold (Fig. 3C), strongly supporting the chondrogenic differentiation of CPCs induced by GDF5.

To further assess matrix production in the 2D system, GAG production was evaluated using DMMB staining, with GAG concentrations normalized to genome copy numbers measured by the PicoGreen assay to account for potential effects of cell proliferation. DMMB is a cationic dye that interacts with sulfate groups presented in GAGs, resulting in a metachromatic change that is dependent on its concentration [30]. As shown in Fig. 3D, GDF5 stimulated modest increases in GAG production in CPC monolayer cultures by day 7 post-treatment, with significant elevations observed at 100 ng/mL and 200 ng/mL of GDF5.

**Fig. 4:**
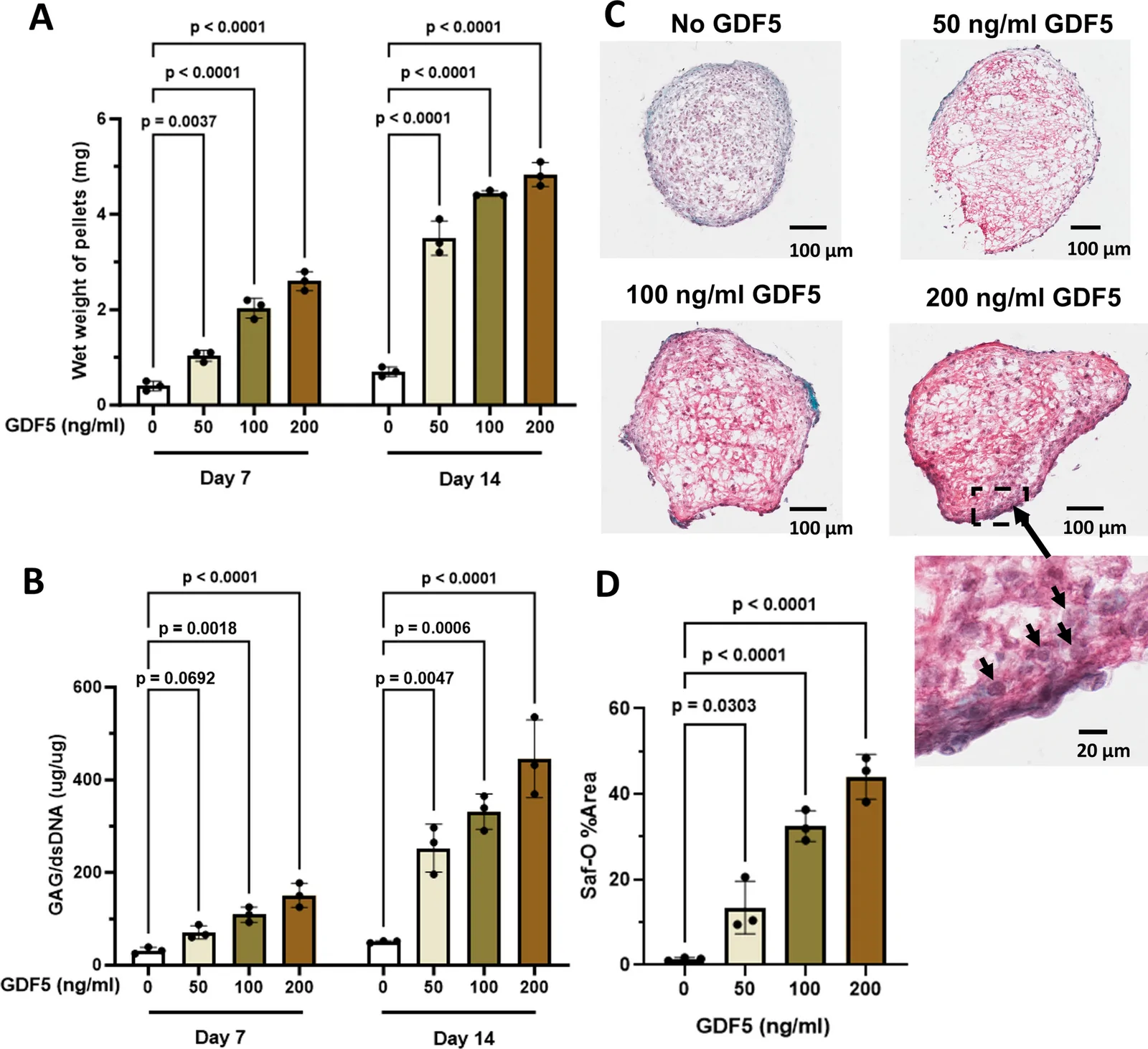
Chondrogenic effects of GDF5 on CPCs in a pellet model. (**A**) Wet weight of pellets, (**B**) GAGs production normalized to dsDNA content within the pellets, (**C**) Saf-O/fast green histological staining, with arrows indicating a notable presence of chondrocyte-like cells in the GDF5-treated groups, and (**D**) quantitative analysis of Saf-O staining intensity following GDF5 treatment (*n* = 3). Data are represented as means ± SD. Statistical analysis was performed using one-way ANOVA, followed by Sidak’s post-hoc test. A *p*-value less than or equal to 0.05 was considered significant.

In chondrogenesis studies using a 3D pellet model, wet weight measurements serve as an indirect indicator of tissue growth and ECM deposition within cell pellets [42]. GDF5 treatment led to a substantial, dose-dependent increase in pellet wet weight over the course of the study (Fig. 4A). It reflected the accumulation of newly synthesized matrix components, suggesting successful chondrogenic differentiation and matrix formation.

Similarly, GDF5 significantly enhanced GAG production in 3D pellets in a dose-dependent manner, with the most pronounced effects observed at 200 ng/ml GDF5 (Fig. 4B). This stimulatory effect on GAG production accumulated progressively over the two-week treatment period. Notably, GAG accumulation in the 3D pellet model was markedly greater than in the 2D monolayer system (Fig. 3D), reflecting the enhanced matrix retention capacity of 3D culture formats [30].

To further investigate the effect of GDF5 on chondrogenic differentiation, we performed Saf-O/fast green staining to visualize and quantify proteoglycan formation within the pellets [43]. Microscopic images on day 14 revealed intense red staining in all GDF5-treated groups, indicating elevated proteoglycan production (Fig. 4C). The intensity of the red staining increased proportionally with GDF5 concentration (Fig. 4D). Additionally, histological images showed a notable presence of chondrocyte-like cells in the GDF5-treated groups, as indicated by arrows in Fig. 4C.

Taken together, these results demonstrate that GDF5 treatment induces chondrogenic differentiation *in vitro* by enhancing key chondrogenic biomarkers and promoting GAG production and cartilage matrix formation.

### RNA Sequencing

**Fig. 5:**
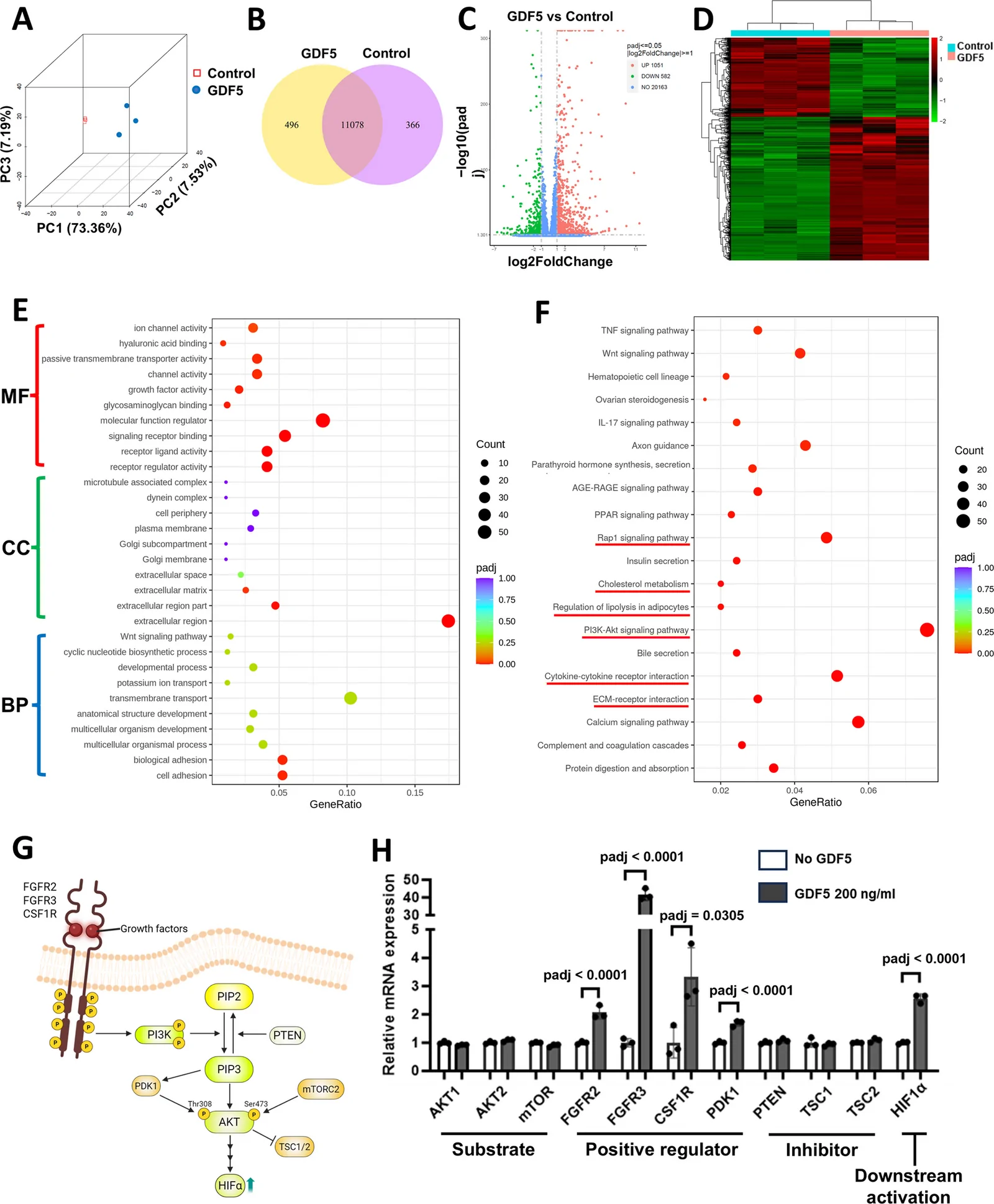
Chondrogenic mechanisms of GDF5 on CPCs via RNA sequencing analysis (*n* = 3). (**A**) Principal component analysis (PCA) 3D plot. (**B**) Venn diagram shows overlap of expressed genes between groups. (**C and D**) DEG analysis between the two groups: (**C**) scatter plot and (**D**) hierarchical clustering. (**E**) GO enrichment (MF: molecular functions, CC: cellular components, BP: biological processes) and (**F**) KEGG enrichment analysis of DEGs. The pathways with red underlines are highly associated with chondrogenic differentiation. (**G**) Schematic of PI3K/Akt pathway in CPC chondrogenic differentiation promoted by GDF5. (**H**) RNA sequencing analysis of PI3K/Akt-related genes reveals no significant changes in substrates and inhibitors, but significant upregulation of positive regulators and downstream effector HIF1α. Data are represented as means ± standard deviation. Differential expression analysis for RNA sequencing was performed using DESeq2, and padj for multiple testing using the Benjamini–Hochberg method to control the FDR. Padj of less than or equal to 0.05 were considered significant. AKT: Protein kinase B, mTOR: Mammalian target of rapamycin, FGFR: Fibroblast growth factor receptors, CSF1R: Colony-stimulating factor 1 receptor, PDK1: Pyruvate Dehydrogenase Kinase 1, PIP2: Phosphatidylinositol 4,5-bisphosphate, PIP3: Phosphatidylinositol (3,4,5)-trisphosphate, PTEN: Phosphatase and tensin homolog, TSC: Tuberous sclerosis complex, HIF1α: hypoxia-inducible factor 1 alpha.

We further analyzed the RNA-seq data of CPCs to explore the mechanism underlying GDF5-induced chondrogenic differentiation. The gene expression profiles of biological replicates within treated and control groups showed high reproducibility, and the principal component analysis revealed a clear distinction between the two groups (Fig. 5A and 5B). Aggregating the replicates for each group, our analysis identified 1,633 differentially expressed genes (DEGs) between the GDF5-treated and control groups, comprising 1,051 upregulated genes and 582 downregulated genes (Fig. 5C and 5D).

The top 30 upregulated and downregulated genes identified from the DEGs ranked by absolute log2 fold change and adjusted *p*-value are presented in Table II and Figure S2. Among the upregulated genes, those related to collagen production, including collagen type II, IX, and XI, were predominant compared to non-treated CPCs. Collagen type II is the primary structural component of cartilage matrix, while collagen type IX and XI are also vital collagens that form a copolymeric network with Type II collagen to provide stability and regulate fibril structure [44, 45]. Specifically, type XI collagen likely copolymerizes with type II collagen, whereas type IX collagen decorates the fibril surface [44, 45]. Together, they reinforce the cartilage extracellular matrix and preserve its biomechanical properties against mechanical stress. These findings highlight that GDF5 enhances chondrogenesis of CPCs by reinforcing both major and minor collagen networks critical for cartilage homeostasis.

**Table II:** List of Significantly Up/down-regulated mRNAs (mRNA: ≥ 0.01% Population)

GDF5-treated CPCs *versus* non-treated CPCs					
Up-regulation			Down-regulation		
mRNA	Log2 (fold change)	Adjusted *p* value	mRNA	Log2 (fold change)	Adjusted *p* value
COL9A3	10.73	< 0.0001	VCAN	−2.92	< 0.0001
COL9A2	9.70	< 0.0001	IGFBP3	−2.82	< 0.0001
COL11A2	9.37	< 0.0001	NBL1	−2.25	< 0.0001
COL2A1	8.72	< 0.0001	DPYSL3	−2.09	< 0.0001
COL9A1	6.72	< 0.0001	CDH2	−2.01	< 0.0001
MXRA5	6.28	< 0.0001	ADAMTS5	−1.77	< 0.0001
MELTF	5.58	< 0.0001	VDR	−1.65	< 0.0001
APOA1	5.44	< 0.0001	ADAMTS1	−1.63	< 0.0001
ANXA8L1	5.37	< 0.0001	SEMA3B	−1.60	< 0.0001
GFAP	5.30	< 0.0001	TGFB2	−1.60	< 0.0001
HAPLN1	5.16	< 0.0001	IFIT1	−1.58	< 0.0001
ACAN	4.96	< 0.0001	SEMA3D	−1.49	< 0.0001
C1QTNF3	4.51	< 0.0001	PRSS35	−1.46	< 0.0001
POSTN	3.85	< 0.0001	MMP14	−1.44	< 0.0001
CHAD	3.84	< 0.0001	GEM	−1.40	< 0.0001
PTH1R	3.67	< 0.0001	SHF	−1.34	< 0.0001
SP7	3.60	< 0.0001	PLXNA2	−1.33	< 0.0001
WWP2	3.42	< 0.0001	CTSV	−1.30	< 0.0001
APOD	3.33	< 0.0001	RNF213	−1.25	< 0.0001
PAPSS2	3.32	< 0.0001	MARCKS	−1.25	< 0.0001
CILP2	2.92	< 0.0001	NIBAN1	−1.25	< 0.0001
COMP	2.84	< 0.0001	GLIS2	−1.24	< 0.0001
SCD	2.77	< 0.0001	LTBP3	−1.22	< 0.0001
SERPINE1	2.68	< 0.0001	LGR4	−1.22	< 0.0001
ANGPTL2	2.67	< 0.0001	ANTXR1	−1.16	< 0.0001
CHST6	2.56	< 0.0001	FIBIN	−1.15	< 0.0001
SOX9	2.53	< 0.0001	HYOU1	−1.13	< 0.0001
F13A1	2.50	< 0.0001	NCAM1	−1.11	< 0.0001
S100B	2.49	< 0.0001	SEPTIN11	−1.10	< 0.0001
IER3	2.42	< 0.0001	TP53INP1	−1.10	< 0.0001

In Table II, other genes related to ECM production, such as *HAPLN1* (5.16-times higher Log2 *versus* control), *ACAN* (4.96-times higher Log2 *versus* control), and *COMP* (2.84-times higher Log2 *versus* control), as well as *SOX9* (2.53-times higher Log2 *versus* control), a key regulator of chondrogenesis, were significantly upregulated following GDF5 treatment. Additionally, several other highly upregulated genes have been implicated in chondrogenic differentiation. These include matrix remodeling-associated 5 (*MXRA5*; 6.2-times higher Log2 *versus* control), which plays a role in maintaining chondrocyte integrity and regeneration [46]; Melanotransferrin (*MELTF*; 5.58-times higher Log2 *versus* control), which has been associated with chondrogenic differentiation in mouse pluripotent mesenchymal C3H10T1/2 cells and prechondrogenic ATDC5 cells *in vitro* [47]; and Apolipoprotein A1 (*ApoA-1*; 5.44-times higher Log2 *versus* control), which facilitates cholesterol transport and lipoprotein metabolism during chondrogenic differentiation [48]. In contrast, the downregulated genes were fewer in number and exhibited lower overall Log2 fold changes compared to the upregulated genes. However, several downregulated genes, such as *ADAMTS1* (1.63-times lower Log2 *versus* control), *ADAMTS5* (1.77-times lower Log2 *versus* control), and *MMP14* (1.44-times lower Log2 *versus* control), were identified as regulators of ECM catabolic enzymes [6, 12, 49, 50]. This suggests that GDF5-dependent ECM generation may partly occur through the suppression of enzymes that degrade the ECM.

The GO enrichment analysis, which interprets the biological significance of differentially expressed genes based on their association with molecular functions (MF), cellular components (CC), and biological processes (BP), highlighted the top 30 most significant terms in each category (Fig. 5E). In the MF subclass, terms related to cell surface receptor signaling were significantly enriched, including molecular function regulator, signaling receptor binding, and receptor regulator/ligand activity. For the CC subclass, DEGs were strongly associated with cartilage ECM organization, as reflected in terms like extracellular region and ECM. In the BP subclass, GDF5 treatment significantly enriched cell adhesion and biological adhesion. These enriched terms play pivotal roles in the chondrogenic differentiation of stem cells. Cell adhesion supports proper condensation and differentiation into chondrocytes, while cell surface receptor signaling enables the reception of external cues, such as growth factors, to activate pathways that drive the transformation of stem cells into cartilage-producing chondrocytes [51, 52]. Differentiated chondrocytes then contribute to ECM formation by secreting essential molecules.

To further explore the molecular pathways influenced by GDF5, we conducted KEGG enrichment analysis (Fig. 5F). The results suggested that functional pathways associated with phosphatidylinositol 3-kinase (PI3K)/protein kinase B (Akt) signaling, Ras-proximate-1 (Rap1) signaling, lipid metabolism, cytokine-cytokine receptor interaction, and ECM-receptor interaction relevant to chondrogenic differentiation were significantly enriched. GSEA further verified that GDF5 treatment was positively related to PI3K/Akt signaling, fatty acid metabolism, and ECM receptor interaction pathways (Figure S1B-S1D).

Rap1 signaling plays a pivotal role in various cellular processes, including cell adhesion, migration, and the regulation of the PI3K/Akt signaling pathway [53]. The PI3K/Akt pathway is a well-known positive regulator of chondrogenic differentiation and plays a chondroprotective role by promoting chondrocyte survival, proliferation, and ECM synthesis [54–56]. This pathway (Fig. 5G) is initiated by the activation of receptors such as fibroblast growth factor receptor 2 (FGFR2), FGFR3, or colony stimulating factor 1 receptor (CSF1R), which stimulate PI3K to convert phosphatidylinositol 4,5-bisphosphate (PIP2) into PIP3 at the cell membrane. PIP3 then recruits 3-phosphoinositide-dependent protein kinase-1 (PDK1) to phosphorylate AKT at Thr308, activating it. Activated AKT phosphorylates downstream targets, including mammalian target of rapamycin complex 1 (mTORC1), which subsequently drives chondrocyte differentiation and ECM production [56]. We compared key genes in the PI3K/Akt pathway between control and GDF5-treated groups based on DEGs (Fig. 5H). While the messenger RNA (mRNA) levels of core substrates, such as AKT1, AKT2, and mTOR, showed no notable changes, there was a significant upregulation of upstream regulators and signaling receptors, including FGFR2, FGFR3, CSF1R, and PDK1. In contrast, key negative regulators of the pathway, including phosphatase and tension homolog (PTEN) (which dephosphorylates PIP3 to suppress AKT activation) and tuberous sclerosis 1 or 2** (**TSC1 or TSC2, which inhibits mTORC1) [56], also showed no change in expression levels. These findings suggest that the significant upregulation of FGFR2, FGFR3, and CSF1R amplifies extracellular signals in response to GDF5 treatment, activating downstream regulators like PDK1 and enabling AKT phosphorylation. This cascade promotes the expression of chondrogenic markers, such as SOX9, COL2a1, and ACAN, which are critical for cartilage matrix synthesis and cell differentiation.

The PI3K/Akt pathway has been reported to activate the transcription and translation of hypoxia-inducible factor 1 alpha (HIF1α), a key regulator of chondrogenesis under hypoxic conditions [57]. Consistent with this, our results led to a significant upregulation of HIF1α mRNA level (Fig. 5H) and enrichment of the HIF-1 signaling pathway as indicated by GSEA (Figure S1E). HIF1α supports the development of chondrocytes and cartilage in part by regulating SOX9 expression [57, 58], and also drives metabolic reprogramming toward glycolysis, meeting the energy demands required during proliferation and differentiation [57, 59]. This aligns with the observation in our proliferation assays, where GDF5-treated CPCs exhibited a shift to glycolytic metabolism and enhanced proliferation. Since ATP production via oxidative phosphorylation is limited in hypoxic environments, the induction of glycolysis through HIF1α serves as a hallmark of CPCs differentiating toward a chondrocyte-like phenotype [38, 39, 58]. Overall, the upregulation of HIF1α induced by GDF5 through the PI3K/Akt-HIF1α axis appears to coordinate both glycolytic activation and chondrogenic lineage commitment.

### Inhibition of ADAMTS5

**Fig. 6:**
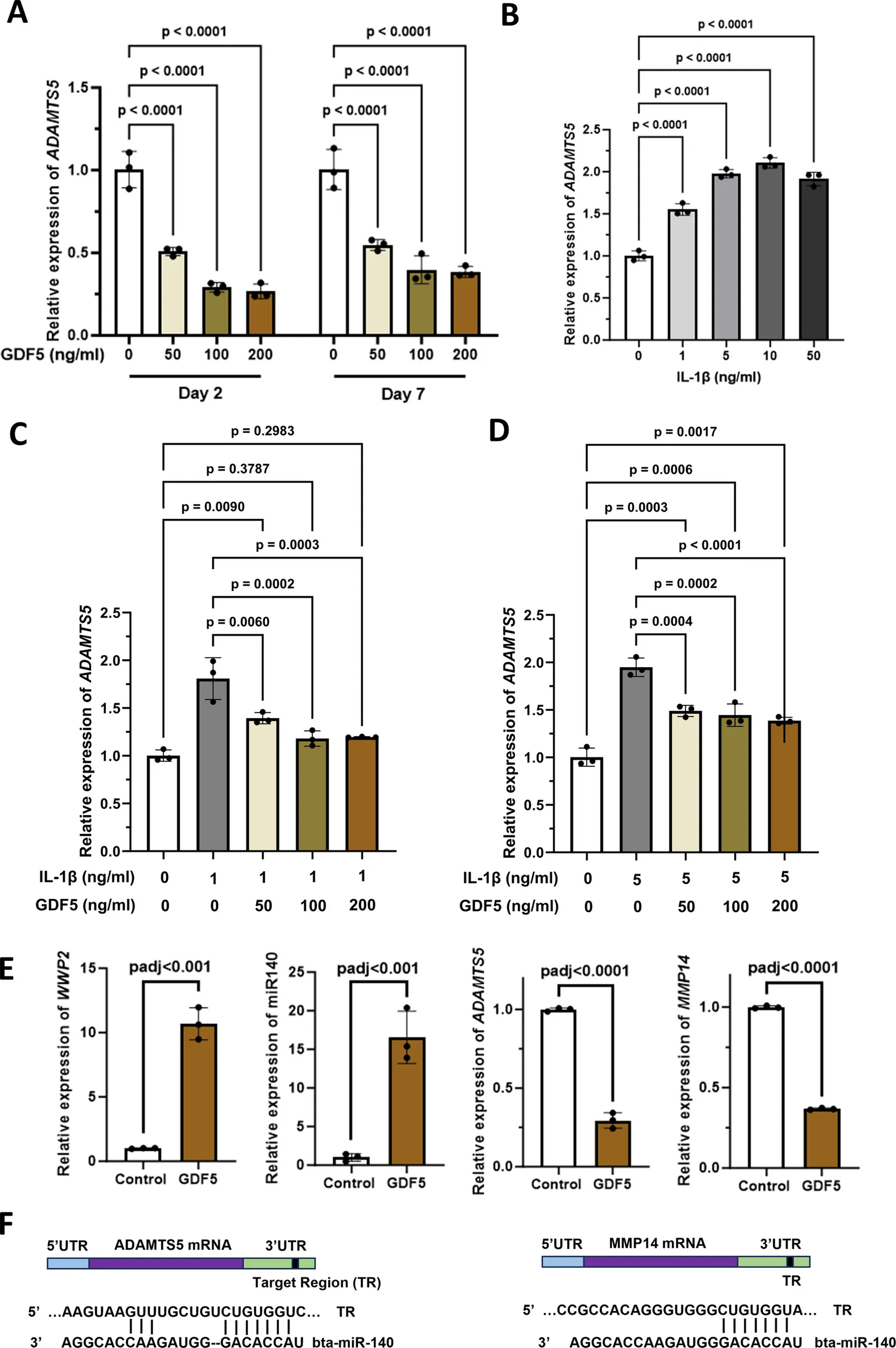
GDF5 reduces the expression of *ADAMTS5* under both normal and inflammatory conditions. (**A-D**) qRT-PCR analysis of *ADAMTS5* in a CPC monolayer culture model (*n* = 3): (**A**) under normal conditions following treatment with GDF5, (**B**) dose-dependent response of *ADAMTS5* expression in CPCs exposed to indicated concentrations of IL1β, with 1 ng/ml and 5 ng/ml selected for subsequent experiments in inflammatory conditions (*n* = 3), and under inflammatory conditions with (**C**) 1 and (**D**) 5 ng/ml IL-1β after GDF5 treatment. (**E**) RNA sequencing analysis of *WWP2, miR140*, *ADAMTS5*, and *MMP14* after GDF5 treatment (*n* = 3). (**F**) miR140 binding sites in the 3’-UTRs of *ADAMTS5* and *MMP14*. Data are represented as means ± standard deviation. Statistical analysis was performed using one-way ANOVA, followed by Sidak’s post-hoc test. A *p*-value less than or equal to 0.05. Differential expression analysis for RNA sequencing was performed using DESeq2, and padj for multiple testing using the Benjamini–Hochberg method to control the FDR. Padj of less than or equal to 0.05 were considered significant.

In the RNA sequencing results, GDF5 treatment induced a significant downregulation of *ADAMTS5* (1.77-times lower Log2 *versus* control), which is a typical aggrecanase and a key contributor to articular cartilage breakdown and matrix degradation [6, 20]. This finding was further validated using qRT-PCR, as shown in Fig. 6A. In the 2D monolayer cultures, GDF5 treatment demonstrated a dose-dependent reduction in *ADAMTS5* expression levels on both day 2 and day 7, with significant inhibition observed.

To assess the protective role of GDF5 in CPCs under inflammatory conditions, the optimal dose of rbIL-1β was determined *in vitro*. As shown in Fig. 6B, *ADAMTS5* expression was significantly elevated following treatment with 1 ng/ml rbIL-1β, with 5 ng/ml inducing a plateau for maximal transcriptional activation. These concentrations (1 and 5 ng/ml) were subsequently used for further experiments. After two days of GDF5 treatment, CPCs were exposed to rbIL-1β for 24 h, and *ADAMTS5* expression was analyzed via qRT-PCR. GDF5 significantly suppressed IL-1β-induced *ADAMTS5* expression in a dose-dependent manner at both 1 and 5 ng/ml of rbIL-1β (Fig. 6C and 6D). Notably, the substantial upregulation of *ADAMTS5* induced by 1 ng/ml rbIL-1β was completely reversed by GDF5 treatment (100 and 200 ng/ml), with no significant difference observed between the untreated group and the GDF5 + rbIL-1β groups (Fig. 6C).

Currently, no direct mechanism has been reported for ADAMTS5 inhibition by GDF5. To explore potential pathways, we examined the significantly upregulated genes in the DEGs from RNA sequencing and found that WW domain containing E3 ubiquitin protein ligase 2 (*WWP2*) was elevated approximately 10-fold following GDF5 treatment in CPCs (Fig. 6E). Previous studies have demonstrated a connection between WWP2 and ADAMTS5, particularly through microRNA-140 (miR-140), whose host gene is WWP2 [60, 61]. Among the over 20,000 genes detected in our RNA sequencing, 267 miRNAs were observed, with miR-140 showing the most significant upregulation (~ 15-fold) after GDF5 treatment (Fig. 6E). MiR-140 is a well-known regulator of cartilage development and homeostasis, and its high expression during chondrogenesis is consistent with our findings in CPCs [61, 62]. It directly binds to the 3' untranslated region (3' UTR) of ADAMTS5 mRNA, suppressing its expression [62]. The inhibition of *ADAMTS5* by 200 ng/mL GDF5 observed in RNA sequencing was consistent with qRT-PCR results (Fig. 6E), and the predicted miR-140 binding site on the *ADAMTS5* 3' UTR was confirmed using TargetScan (Fig. 6E). Additionally, in the MMPs family, RNA sequencing revealed significant downregulation of *MMP14*, another direct target of miR-140, as predicted by TargetScan (Fig. 6F). This finding indirectly supports the hypothesis that GDF5 inhibits *ADAMTS5* in CPCs through the upregulation of miR-140. Together, these results suggest that GDF5 exerts its chondroprotective effects via the WWP2-miR-140 axis, leading to the suppression of catabolic enzymes such as ADAMTS5 and MMP14.

## Conclusion

This study investigated the roles of GDF5 in supporting CPC-mediated cartilage regeneration. Overall, GDF5 demonstrated multifaceted regenerative roles: it acted as an excellent chemoattractant to guide CPCs toward injury sites when delivered within an HA/fibrin IPN hydrogel, enhanced CPC proliferation to expand the reparative cell pool, and induced the chondrogenic differentiation of CPCs by activating key signaling pathways and enhancing the expression of essential ECM components. In addition, GDF5 suppressed the expression of *ADAMTS5*, a major ECM-degrading enzyme, potentially contributing to matrix preservation and stabilization. These combined effects highlight GDF5 as a promising therapeutic candidate for enhancing CPC-based cartilage regeneration. Future work will focus on utilizing engineered IPN hydrogel platforms to achieve controlled, localized GDF5 delivery to improve CPC-mediated cartilage repair and prevent PTOA progression.

## Supplementary Information

Below is the link to the electronic supplementary material.

ESM 1 (DOCX 691 KB)

## Data Availability

Data will be made available on request.
