# Supplementary material for: The Versatile Role of GDF5 in Chondrogenic Progenitor Cell-mediated Cartilage Regeneration via a Hyaluronic Acid-fibrin IPN Hydrogel Platform
Source: Pharm Res. 2026 Apr 14;43(7):2167–84. doi: 10.1007/s11095-026-04079-3 (PMC13545135; doi:10.1007/s11095-026-04079-3)
Supplement: Supplementary file 1 [file 11095_2026_4079_MOESM1_ESM.docx]

**Supplementary Information**

**The Versatile Role of GDF5 in Chondrogenic Progenitor Cell-Mediated Cartilage Regeneration via a Hyaluronic Acid-Fibrin IPN Hydrogel Platform**

Rui He^1^, Venkateswaran Ganesh^2,3^, Pornpoj Phruttiwanichakun^1^, James A. Martin^1,2,3^, Dongrim Seol^2,3,4^, Aliasger K. Salem^1^*

^1^Pharmaceutical Sciences and Experimental Therapeutics, College of Pharmacy, University of Iowa, Iowa City, IA, USA;

^2^Department of Orthopedics and Rehabilitation, Carver College of Medicine, University of Iowa, Iowa City, IA, USA;

^3^Roy J. Carver Department of Biomedical Engineering, College of Engineering, University of Iowa, Iowa City, IA, USA;

^4^Department of Orthodontics, College of Dentistry and Dental Clinics, University of Iowa, Iowa City, IA, USA

*Correspondence: Aliasger K. Salem (aliasger-salem@uiowa.edu); Pharmaceutical Sciences and Experimental Therapeutics, College of Pharmacy, University of Iowa, 180 S Grand Ave, Iowa City, IA 52242, USA; +1-319-335-8810

**Figure S1**. GSEA enrichment analysis of the glycolysis pathway, PI3k-AKT signaling pathway, fatty acid metabolism pathway, ECM receptor interaction pathway, and the HIF-1 signaling pathway between the GDF5-treated and control groups (200 ng/ml).

**Figure S2.** Heatmap of the top 30 upregulated and downregulated genes identified from the DEGs between the control and GDF5-treated groups (200 ng/ml).
